# Editorial: Social Inequality in Cancer Screening

**DOI:** 10.3389/fpubh.2022.854659

**Published:** 2022-04-28

**Authors:** Guido Van Hal, Hajo Zeeb, Harry J. de Koning

**Affiliations:** ^1^Social Epidemiology and Health Policy, University of Antwerp, Antwerp, Belgium; ^2^Leibniz Institute for Prevention Research and Epidemiology (LG), Berlin, Germany; ^3^Department of Public Health, Erasmus Medical Center, Rotterdam, Netherlands

**Keywords:** cancer screening, inequality, breast cancer screening, cervical cancer screening, colorectal cancer screening, lung cancer screening, socioeconomic status, informed decision

It is generally known there is a so-called social gradient regarding health and health care, meaning that people who are worst off socially and financially also have a worse health and do not get all the health care needed (1). Unfortunately, this link is also present regarding preventive health care. Several studies found that people with a low(er) socio-economic status (SES) take less part in cancer screening and consequently often present with late cancer diagnosis leading to a higher mortality rate, as is also shown in the contribution to this Research Topic from Nuche-Berenguer et al. Moreover, people with a lower SES tend to adhere less to follow-up colonoscopy after a positive Fecal Immunichemical Test (FIT) in colorectal cancer screening (2). However, there is substantial evidence that screening programmes for breast, cervical, colorectal and recently also lung cancer lead to a decrease in cause-specific mortality (3–6). The lower participation rate of specific subgroups in society is worrisome for several reasons. First of all, oftentimes people with a lower SES also have a higher risk of getting cancer. This is the case for colorectal cancer and cervical cancer (7, 8). This also raises an ethical point: can we accept that subgroups in society have a higher risk of dying from cancer, while it is preventable?

It is therefore important to have access to data on the participation in cancer screening from different subgroups in society; to analyse and interpret these data; to develop interventions to increase the participation rate in those subgroups participating less and; to evaluate these interventions.

The articles published in this Research Topic show that these issues are not restricted to a specific region or continent. It contains contributions from the United States over Argentina to the Basque Country and Flanders.

Knowledge about the factors related to non-participation is important. Risk factors for underscreening which are often found, are: low SES, migration background, being un- and under-insured, low health literacy.

Besides increasing knowledge on risk factors, it is also time to look at possible interventions and campaigns to do something about it. In the contribution to the Research Topic of Solis-Ibinagagoitia et al., the authors promote the involvement of primary care workers to improve not only the adherence to colorectal cancer screening but also to other preventive interventions and healthy life styles.

The paper by Guerra et al. applied the Plan-Do-Study-Act (PDSA) quality improvement framework to the development, implementation, and evaluation of a breast cancer navigation program for un- and under-insured women in the USA. Just as Ding et al., they propose to implement not only the “one-size-fits-all” interventions and messages but to develop tailored interventions for these groups that are hard to reach. This will be even more important when lung cancer screening will be implemented, since smokers are likely to be not as prevention-minded as the average target groups for the already well-established cancer screening programmes for breast, cervix and colorectal cancer (9). An important issue in this respect, is to offer understandable but correct information. Referring to the hesitance against the COVID-vaccination in certain groups, misinformation can have a devastating impact on accepting preventive health care. This is also the case with cancer screening. The drawbacks of cancer screening should not be be swept under the carpet but should not be inflated either. People should be able to make an informed decision based on balanced information. This is especially important when screening programmes are being critizised. This was and still is the case for breast cancer screening (10). For too long, the aim was to recruit as many women as possible, only telling them the positive aspects of participation. Participation rate even became a separate health target in some countries. Sometimes, this could even lead to “emotional blackmail”, when people are told that they should participate in cancer screening to see their children or grandchildren growing up, for instance.

Not informing the target group about the possible harms of cancer screening led to disappointment among participants after a false negative or false positive result, since they were not aware beforehand that this could happen. Although there is nowadays much more attention for giving correct and understandable information to the target group for breast cancer screening, there still is a lot of work to do, also in Europe (11). Fears exist that by not only stressing the benefits but also the possible harms, the participation rate will decrease. However, this concern has been refuted in a number of previous studies (12–17). Nevertheless, in Flanders the strategy of informed choice was changed toward “informed motivation”, meaning that the target group is informed about both the benefits and potential harms but that it is also made clear that the organizing Government believes it is worthwhile to participate (18).

To better involve those who are considered hard-to-reach for cervical cancer screening, self-sampling might be part of the solution. Self-sampling for HPV with a brush showed to be acceptable to Flemish postmenopausal non-responders (19). Also first-void urine can be collected at home by women and was shown as an interesting screening technique to reach non-participants (20). A similar result was found during the pilot project for the Flemish colorectal cancer screening programme. Two invitation strategies for screening for colorectal cancer with the FIT were compared and it became very clear that the participation rate was much higher in the target group receiving the test kit at home compared to the target group that received an invitation to first visit their GP (21).

The important work highlighted in this Research Topic indicates that much has been done already and new insights into screening and social inequality can be gained through deidicated research including new interventions. As new generations enter into the programmes and social inequality remains a central challenge to societies worldwide, further efforts in research and implementaion are required to reduce the social inequality and inequity in cancer screening.

## Author Contributions

GV has written the editorial. HZ and HK have given their comments and suggestions on the text. All authors have read and approved the final version.

## Conflict of Interest

The authors declare that the research was conducted in the absence of any commercial or financial relationships that could be construed as a potential conflict of interest.

## Publisher's Note

All claims expressed in this article are solely those of the authors and do not necessarily represent those of their affiliated organizations, or those of the publisher, the editors and the reviewers. Any product that may be evaluated in this article, or claim that may be made by its manufacturer, is not guaranteed or endorsed by the publisher.
